# The mechanism of all-*trans* retinoic acid in the regulation of apelin expression in vascular endothelial cells

**DOI:** 10.1042/BSR20170684

**Published:** 2017-12-12

**Authors:** Hongyun Shi, Lanhui Yuan, Huibin Yang, Aimin Zang

**Affiliations:** Department of Oncology, The Affiliated Hospital of Hebei University, Baoding 071000, Hebei, China

**Keywords:** ATRA, Apelin, VECs

## Abstract

The *apelin* gene can promote vascular endothelial cell (VEC) proliferation, migration, and angiogenesis. However, the molecular mechanism for regulation of the *apelin* gene is still unknown. Real-time PCR and Western blotting analysis were employed to detect the effect of all-*trans* retinoic acid (ATRA) in up-regulating apelin expression in human umbilical vein endothelial cells (HUVECs). Furthermore, the *in vivo* study also indicated that ATRA could increase apelin expression in balloon-injured arteries of rats, which is consistent with the results from the cultured HUVECs. To ensure whether retinoic acid receptor (RAR) α (RARα) could be induced by ATRA in regulating apelin, the expression of RARα was tested with a siRNA method to knock down RARα or adenovirus vector infection to overexpress RARα. The results showed that ATRA could up-regulate apelin expression time- and dose- dependently in HUVECs. ATRA could induce a RARα increase; however, the expression of RARβ and RARγ were unchanged. The blocking of RARα signaling reduced the response of apelin to ATRA when HUVECs were treated with RARα antagonists (Ro 41-5253) or the use of siRNA against RARα (si-RARα) knockdown RARα expression before using ATRA. In addition, induction of RARα overexpression by infection with pAd-GFP-RARα further increased the induction of apelin by ATRA. These results suggested that ATRA up-regulated apelin expression by promoting RARα signaling.

## Introduction

Abnormal proliferation and anti-apoptosis of vascular endothelial cells (VECs) is a key event in cardiovascular disease occurrence and development, such as hypertension, atherosclerosis, and restenosis etc. Apelin is a bioactive peptide that regulates blood pressure, enhances myocardial contraction, and promotes neovascularization by binding to its specific receptor APJ (apelin receptor, aplnr) [1–3]. To date, it has been shown that apelin is expressed in the heart, endothelium, vascular smooth muscle cells, brain, kidney, testis, ovary, liver, and adipose tissue, with the highest levels expressed in the lung and the mammary gland [4,5]. Apelin is secreted as a 77-amino acid preproprotein that is cleaved to form several active peptides denoted by their length as apelin-13, -17, and -36. It has been shown that apelin-13 exhibits much stronger activity than apelin-36 in promoting cell proliferation [6]. Some studies have reported that insulin, TNF-α, all-*trans* retinoic acid (ATRA), and hypoxic conditions may increase apelin expression *in vivo* and *in vitro* [7–12], and some transcription factors may regulate *apelin* gene expression [13,14]. However, the molecular mechanisms inducing apelin expression are still under investigation in the cardiovascular system.

ATRA is a metabolite of dietary vitamin A, which has an effect on the regulation of the growth and inhibition of proliferation in many cell types. Its receptor has retinoic acid receptors (RARs) and retinoid X receptors (RXRs), which belong to the nuclear receptor superfamily and participate in cell proliferation, differentiation, migration, apoptosis, and other processes [15–17]. There are three types of RARs: RARα, RARβ, and RARγ. However, the major bioactive effect is caused by RARα. In a previous study, it was confirmed that apelin-13 could promote VEC proliferation, migration, and neointimal hyperplasia [18,19]. Interestingly, why ATRA, an antiproliferative and prodifferentiation factor, induces the expression of apelin, a mitogen for VECs, remains unknown. Thus, the results described the present study for the first time that ATRA up-regulated apelin expression by RARα and further demonstrated the relationship of ATRA and RARα in the retinoid pathway.

## Materials and methods

### Animals

Male Sprague–Dawley (SD) rats were purchased from the Institutional Animal Care and Use Committee of Charles River (Beijing, China, license number: SCXK 2012-0001). The study protocol conformed to the United States NIH guidelines (Guide for the Care and Use of Laboratory Animals (1985), DHEW publication number (NIH) 85–23: Office of Science and Health Reports, DRR/NIH, Bethesda, MD, U.S.A.).

### Cell culture and treatment

Human umbilical vein endothelial cells (HUVECs) were purchased from the Cell Resource Center of Shanghai Institutes for Biological Sciences, Shanghai, China. HUVECs were maintained in Dulbecco’s modified Eagle’s medium (DMEM) supplemented with 10% FBS, 50 μg/ml endothelial cell growth supplement, 1% glutamine, 100 U/ml penicillin, and 100 μg/ml streptomycin in a humidified atmosphere with 5% CO_2_ at 37°C. Prior to ATRA stimulation, HUVECs were maintained in serum-free DMEM for 24 h. They were then cultured in DMEM containing 5% FBS and 10 μM ATRA (Sigma–Aldrich) for the indicated times. To induce inhibitor Ro 41-5253, cells were pretreated with the indicated inhibitor at a final concentration of 20 μM for 2 h before the addition of 10 μM of ATRA.

### Balloon-injury model

Male SD rats were maintained under a standardized condition. Then, the rats (weighing 300–350 g) were randomly divided amongst three groups (*n*=6): control group (Con + Veh), balloon-injured arteries group (M + Veh), and ATRA treatment group (M + ATRA). The rats were anesthetized with ketamine (60 mg/kg of body weight) and xylazine (5 mg/kg of body weight) intraperitoneally. Briefly, after a median incision on the anterior neck, the carotid arteries of the left side were isolated and a distal incision was made. After the blood was removed, a 0.13-mm diameter balloon catheter was advanced gently into the left common carotid artery. The balloon was inflated with saline to distend the common carotid artery and then pulled back to the external carotid artery. The catheter was withdrawn and the proximal end of the external carotid artery was ligated, and blood flow was re-established after removing the clamps on the arteries. All procedures were performed by a single operator. ATRA was administered orally at a dose of 1 mg/kg of body weight per day beginning 1 day before balloon injury and continuing for 14 days thereafter. On day 14 after injury and administration of ATRA orally, the rats were killed by exsanguination under anesthesia, and the carotid artery was collected for quantitative reverse-transcription PCR (qRT-PCR) or immunohistochemistry.

### Hematoxylin–Eosin staining

Artery tissues of rats were fixed with 10% neutral buffered formalin at room temperature (RT) for 24 h and then embedded in paraffin and sectioned to a thickness of approximately 4 μm. The artery sections were stained with HE following the standard protocol and then light microscopic examination was performed.

### Immunohistochemistry

Artery tissues of rats were fixed with 10% neutral buffered formalin at RT for 24 h, embedded in paraffin, and sectioned to a thickness of approximately 4 μm. The artery sections were stained with immunostaining following the standard protocol. Immunostaining of sections was performed with anti-apelin antibody (1:100 dilution), and these sections were also counterstained with Hematoxylin. Staining intensities were determined by measurement of the integrated optical density (IOD) with light microscopy using the computer-based Image-Pro Morphometric System in a double-blind manner.

### Morphometric analysis of neointima formation

At 14 days after balloon injury, arteries were collected and embedded in paraffin to prepare cross-sections. Neointima thickening was assessed using the intima-to-media (I/M) area ratio measured from Hematoxylin and Eosin-stained arterial cross-sections with the computer-based Image-Pro Morphometric System in a double-blind manner. Four discontinuous sections from each vessel were measured in each SD rat, and four rats were used in each experimental group.

### RNA preparation and qRT-PCR

Total RNA was isolated with TRIzol reagent (Invitrogen) according to the manufacturer’s instructions. As an internal control, glyceraldehyde-3-phosphate dehydrogenase (*GAPDH*) gene primers were used for RNA template normalization. Quantitative PCR of apelin was performed using a Platinum SYBR Green qPCR SuperMix UDG Kit (Invitrogen). The following primers were used: apelin (rat), 5′-AGACCCCGGAGGCTAAGGAGTT-3′ (sense) and 5′-TCCGTCATAGTGTCCTCCATCA-3′ (antisense); GAPDH (rat), 5′-GCTCTCTGCTCCTCCCTGTT-3′ (sense) and 5′-GTGGCAGTGATGGCATGGAC-3′ (antisense). The relative expression level was calculated using the following equation: relative gene expression = 2^−ΔΔ*C*^_T_.

### Semiquantitative PCR

Total RNA was isolated with TRIzol reagent (Invitrogen) according to the manufacturer’s instructions. RT-PCR was performed. The PCR conditions were 25 cycles at 94°C for 30 s, 55°C for 30 s, and 72°C for 45 s. As an internal control, *GAPDH* gene primers were used for RNA template normalization. The following primers were used: apelin (human), 5′-AGGGCTGCCACGGACCGAGT-3′ (sense) and 5′-CTGGCACCGGGAGGGCACTT-3′ (antisense); GAPDH (Human), 5′-CAGCCCAGAACATCATCCCT-3′ (sense) and 5′-GCCTCTCTCTTGCTCTCAGTA-3′ (antisense).

### Western blotting

Cells were treated with ATRA for various times or at different doses and then harvested with 150 mmol/l NaCl, 50 mmol/l Tris/HCl (pH 7.5), 1% NP-40, 0.5% sodium deoxycholic acid, and complete protease inhibitor mixture tablets (Roche Applied Science, Basel, Switzerland). Crude proteins were isolated from HUVECs, separated by SDS/PAGE, and transferred on to PVDF membranes (Millipore, Billerica, MA, U.S.A.). Membranes were blocked with 5% milk in TBS with Tween 20 (TTBS) for 2 h at 37°C and then incubated overnight at 4°C with the following primary antibodies: 1:500 rabbit anti-apelin (GeneTex, U.S.A.), 1:800 rabbit anti-RARα, 1:800 rabbit anti-RARβ, and 1:800 rabbit anti-RARγ (Abcam), and 1:1000 mouse anti-β-actin (Santa Cruz Biotechnology, CA, U.S.A.). After incubation with the appropriate secondary antibody, the immunoreactive signals of antibody antigens were visualized using the Chemiluminescence Plus Western Blot analysis kit (Santa Cruz Biotechnology).

### siRNA transfection

Nonspecific siRNA (si-NS) and siRNAs specific for human RARα were purchased from Santa Cruz Biotechnology (CA, U.S.A.). Transfection was performed using Lipofectamine reagent (Invitrogen) following the manufacturer’s instructions. Twenty-four hours following transfection, HUVECs were treated with or without ATRA (10 μM). Cells were then harvested and prepared for a Western blotting assay.

### Statistical analyses

Data are presented as bar graphs of the mean ± S.E.M. (S.D.) of ≥3 independent experiments. Statistical analyses were performed using the one-way ANOVA, and post-hoc test according to the number of groups compared. Differences were considered significant at *P*<0.05.

## Results

### ATRA up-regulated apelin transcription and translation in cultured HUVECs

**Figure 1:**
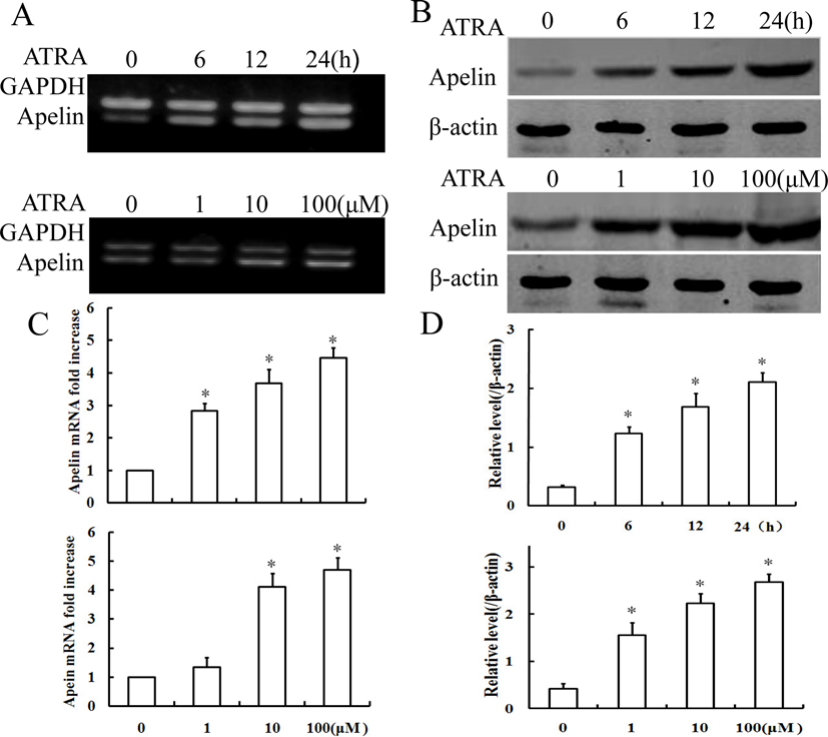
ATRA up-regulated apelin transcription and translation in HUVECs HUVECs were treated with ATRA (10 μΜ) for various times or at different doses (for 24 h). (**A**) Total RNA was transcribed with reverse transcriptase and amplified by PCR. GAPDH was used as an internal control. (**B**) Crude proteins were extracted from the treated cells and then Western blotting was performed using anti-apelin antibodies to examine the expression of apelin. β-actin was used as a loading control. (**C**) The mRNA levels of apelin were determined by qRT-PCR analysis. Bars represent the mean ± S.D. from three independent experiments. *P<0.05 compared with the respective control group. (**D**) Densitometry of (B); results were normalized to β-actin. Bars represent the mean ± S.D. from three independent experiments. *P<0.05 compared with the respective control group.

To validate the effect of apelin expression in response to ATRA signaling, RT-PCR, qRT-PCR, and Western blotting were used to further examine apelin expression in terms of transcription and translation levels. When HUVECs were treated with 10 μΜ ATRA for various times, *apelin* mRNA and protein levels obviously increased in a time-dependent manner. Moreover, ATRA also dose-dependently increased *apelin* mRNA and protein levels (Figure 1A–D).

### ATRA up-regulated apelin expression in injured arteries

**Figure 2:**
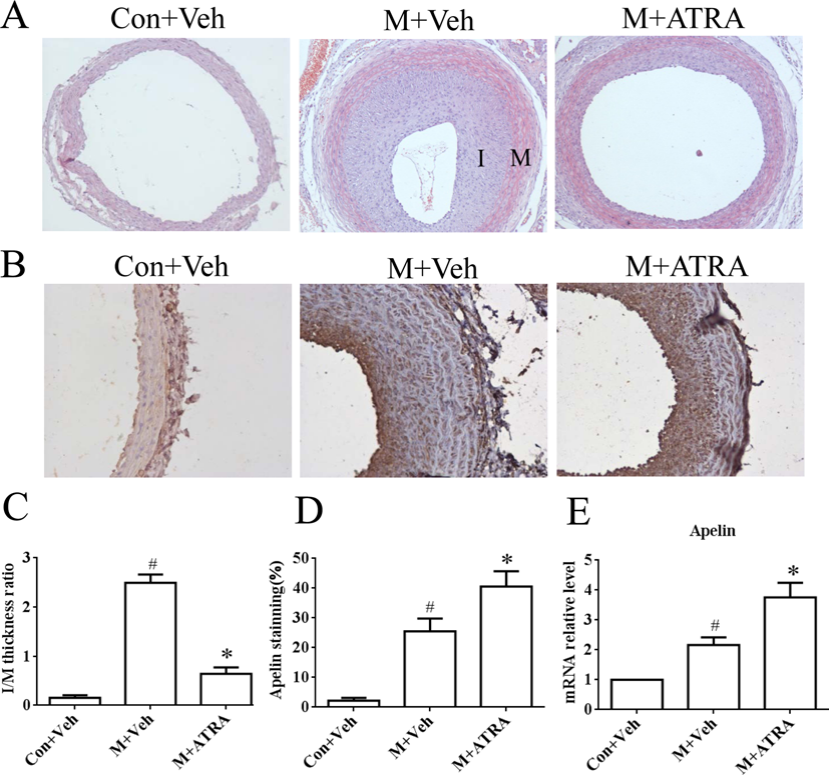
The expression of apelin in balloon-injured arteries (**A**) Representative Hematoxylin and Eosin-stained arterial sections from the control plus vehicle (Veh), model (M) plus vehicle, and model plus ATRA. Magnification: ×100. (**B**) Immunostaining of apelin in carotid arteries on day 14 after balloon injury. Magnification: ×200. (**C**) The ratio of I/M of carotid arteries on day 14 after balloon injury. ^#^*P*<0.01 compared with the control group; *P<0.01 compared with the model group (*n*=6 in each group). (**D**) The apelin index was calculated as follows: apelin-positive cells/total intimal cells. The results are means ± S.D. for four independent experiments. ^#^*P*<0.01 compared with the control group; *P<0.01 compared with model group (*n*=6 in each group). (**E**) The levels of *apelin* mRNA were determined by qRT-PCR analysis. ^#^*P*<0.01 compared with the control group; *P<0.01 compared with the model group (*n*=6 in each group).

To better understand the expression of apelin on the effect of ATRA, we investigated the expression of apelin in the balloon-injured arteries in rats on administration of ATRA. At 14 days after the balloon injury, the model rats showed abundant neointimal hyperplasia, which was significantly lower in ATRA-treated animals. The uninjured arteries of the model group revealed no significant neointimal hyperplasia (Figure 2A,C). The expression of apelin was examined by immunohistochemical staining of the tissue (Figure 2B). The results showed that the uninjured arteries had a low expression of apelin. Balloon injury of the vessels could induce apelin expression in balloon-injured arteries from vehicle-treated rats, where 25.51% of cells were apelin-positive; however, upon ATRA treatment, 40.60% of the cells became apelin-positive within the intimal lesions (Figure 2D). Quantitative real-time PCR analysis showed that ATRA treatment significantly increased *apelin* mRNA expression in balloon-injured arteries compared with control animals (Figure 2E). These results suggested that ATRA increased apelin expression in balloon-injured arteries of rats, which was consistent with the results from the cultured HUVECs.

### The expression of RARα, RARβ, or RARγ was detected by ATRA

**Figure 3:**
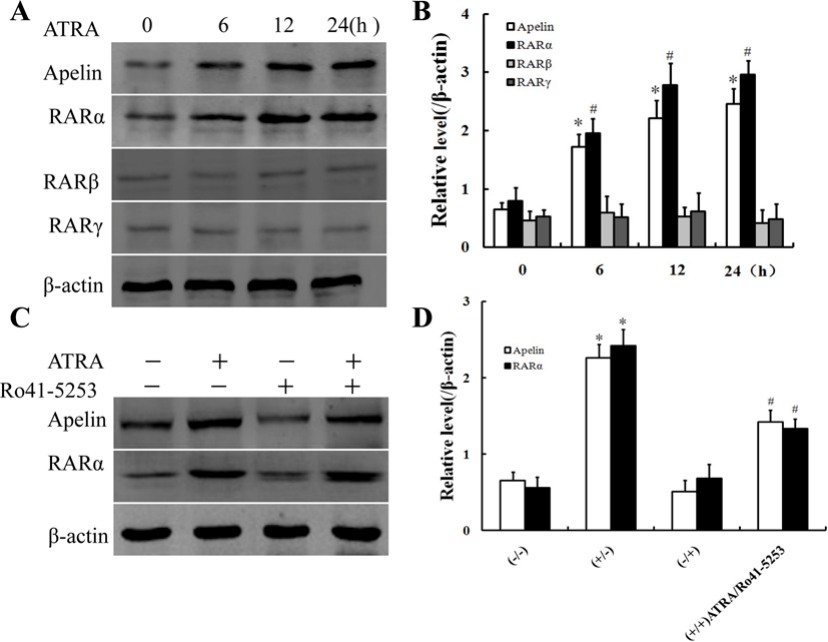
RARα was detected and Ro 41-5253 reduced the response of apelin to ATRA (**A**) HUVECs were treated with 10 μM of ATRA for 0, 6, 12, or 24 h. Crude proteins were extracted from the treated cells and then subjected to Western blotting with the antibodies against apelin, RARα, RARβ, and RARγ respectively. β-actin was used as a control for equal protein loading. (**B**) Densitometry of (A); results were normalized to β-actin. Bars represent the mean ± S.D. from three independent experiments. *,#*P*<0.05 compared with ATRA-free group. (**C**) HUVECs were pretreated with 20 μM of Ro 41-5253 for 2 h prior to exposure to ATRA (10 μM) for 24 h. Crude proteins from cell lysates were analyzed by Western blotting with the antibodies against apelin and RARα, respectively. β-actin was the loading control. (**D**) Densitometry of (C); results were normalized to β-actin. Bars represent the mean ± S.D. from three independent experiments. *P<0.05 compared with ATRA-free group. ^#^*P*<0.05 compared with ATRA-treated group.

There are three types of RARs: RARα, RARβ, and RARγ, each of which is encoded by their respective genes. To further define which RAR isoform mediated the induction of apelin by ATRA, HUVECs were treated with 10 μΜ ATRA for various times. As shown in Figure 3A,B, ATRA markedly increased apelin and RARα expressions in a time-dependent manner. However, unlike RARα, the expressions of RARβ and RARγ were little changed by the ATRA treatment. Collectively, these results suggested that RARα, but not RARβ or RARγ, was essential for induction of apelin expression by ATRA.

### Ro 41-5253 reduced the response of apelin to ATRA

Several studies have reported that ATRA produces its regulatory effect via retinoic acid response element (RARE) or a RARE-independent manner. To explore whether ATRA up-regulates apelin expression by RARα, HUVECs were treated with a RARα antagonist (Ro 41-5253) prior to the addition of ATRA. The results showed that the blockade of RARα signaling partially reduced the response of apelin to ATRA (Figure 3C,D).

### The effect of ATRA on the expression of apelin was detected by knockdown or overexpression of RARα

**Figure 4:**
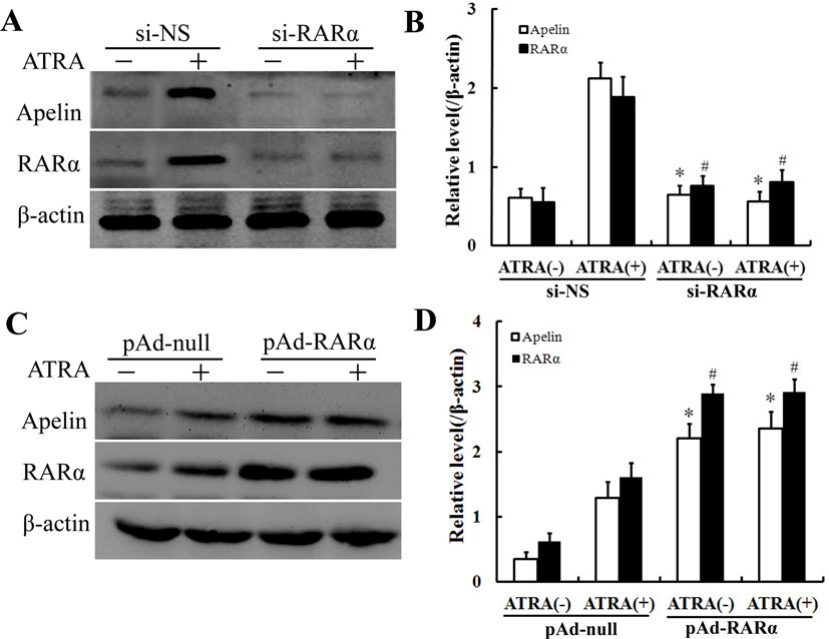
The effect of ATRA on the expression of apelin was detected by knockdown or overexpression of RARα (**A**) HUVECs were transfected with si-RARα or si-NS for 24 h and then treated with 10 μM of ATRA for 24 h. The cell lysates were analyzed by Western blotting with the antibodies against apelin and RARα, respectively. β-actin was the loading control. (**B**) Densitometry of (A); results were normalized to β-actin. Bars represent the mean ± S.D. from three independent experiments. *^,#^P<0.05 compared with si-NS group. (**C**) HUVECs were infected with pAd-null or pAd-GFP-RARα for 24 h prior to the exposure to ATRA (10 μM) for 24 h. Crude proteins from cell lysates were analyzed by Western blotting with antibodies against apelin or RARα. β-actin was the loading control. (**D**) Densitometry of (C); results were normalized to β-actin. Bars represent the mean ± S.D. from three independent experiments. *^,#^*P*<0.05 compared with pAd-null group.

To further test whether RARα mediated the induction of apelin by ATRA, we knocked down endogenous RARα by transfecting HUVECs with siRNA against RARα (si-RARα) or a control (si-NS), respectively. The induction of apelin by ATRA was significantly reduced at a protein level in the cells with RARα knockdown (Figure 4A,B). Moreover, RARα overexpression introduced by infection of the adenovirus vector pAd-GFP-RARα, further increased the induction of apelin by ATRA (Figure 4C,D).

## Discussion

Apelin is distributed in diverse peripheral organ tissues and plays various roles in the physiology and pathophysiology of many organs. Apelin participates in the regulation of blood pressure, new blood vessel formation, and cardiac function ischemia–reperfusion injury and atherogenesis in regulating cardiovascular actions [20–23]. However, apelin could play a dual function in regulating blood pressure or atherogenesis. For example, previous physiological experiments in anesthetized and conscious animal models have yielded conflicting results about the role of apelin in regulating vascular tone and blood pressure. In anesthetized and conscious rats, systemic administration of apelin resulted in a transient drop in systolic and diastolic blood pressure with no change in heart rate [24]. However, the study reported that *apelin* mRNA and protein expressions were markedly depressed in spontaneously hypertensive rats (SHR) [10] The new study confirmed that Elabela/Toddler expression, as an endogenous agonist of the APJ receptor, was reduced in cardiopulmonary tissues from pulmonary arterial hypertension (PAH) patients and PAH rat models [25]. These results suggested that apelin, in regulating cardiovascular actions, had a biphasic effect on blood vessels. Also, up-regulation of apelin promoted neointima formation in the carotid ligation model in mice [26]. However, in ApoE-deficient mice, apelin infusion inhibited atherogenesis and completely abrogated angiotensin II-accelerated atherosclerosis [27]. These data suggested that apelin also had a biphasic effect on the progression of atherosclerosis.

The studies have reported that insulin, TNF-α, and hypoxic conditions may increase apelin expression *in vivo* and *in vitro*. Intriguingly, apelin expression had different expression levels with the same stimulant in different tissues or cells. For example, Daviaud et al. [28] reported that TNF-α up-regulated apelin expression in human and mouse adipose tissue, while Melgar-Lesmes [29,30] revealed that TNF-α decreased apelin/APJ expression in hepatic stellate cells (HSCs) but increased apelin expression in HepG2 cells. To validate the effect of apelin expression in response to ATRA signaling in VECs, the *in vitro* and *in vivo* experiments were processed. From Figure 1A–D, the expression of apelin in terms of transcription and translation was increased in a time- and dose-dependent manner in ATRA-treated HUVECs. Also, the expression of apelin in the balloon-injured rats on administration of ATRA was increased compared with the other two groups (Figure 2B–D). These results confirmed that ATRA could increase apelin expression in the vessels *in vitro or in vivo*.

ATRA participated in various cell activities such as differentiation, proliferation, migration, apoptosis, or inflammation. It could directly transactivate downstream target genes by binding to RARs or RXRs and inhibited VEC proliferation. RARs bound to RAREs and recruited a protein complex to activate transcription. In addition, several studies have reported that RARs could exert its effect via RARE-independent regulatory mechanisms by interacting with other transcription factors [31,32]. There were three types of RARs: RARα, RARβ, and RARγ, each of which was encoded by their respective genes. The present study was the first report that revealed that RARα, but not RARβ or RARγ, was essential for induction of apelin expression by ATRA in HUVECs. The selective activation of these receptors resulted in distinct biological effects on different cell types. In the present study, we characterized the regulation of apelin through RARα. We discovered that treatment with ATRA induced the expressions of apelin and RARα in a time-dependent manner in HUVECs. However, the expressions of RARβ and RARγ were little changed by the ATRA treatment (Figure 3A,B). Another, knockdown *RARα* gene with si-RARα or inhibiting RARα with its antagonist Ro 41-5253 decreased the ATRA-induced apelin expression (Figures 3C and 4A), indicating that RARα played a critical role in the induction of apelin by ATRA and could not be substituted by other RARs.

Interestingly, why did ATRA, a prodifferentiation factor, induce the expression of apelin, a proliferative factor for VECs? We speculated that preproapelin and its active forms (apelin-36 and apelin-13) might have distinct biological activities necessary to regulate proliferation and differentiation in VECs. The new study verified that APJ activation by all apelin isoforms likely hinges on shared conformation and dynamics in the C-terminus, but bioactivity of the putative apelin proprotein expanded the repertoire of apelin receptor ligands [33]. From supplemental data (Supplementary Figures S1A–D and S2A–D), apelin-13 induced HUVEC proliferation in a time- and dose-dependent manner, and the expression of the proliferation-marked gene *PCNA* and *cyclinD1* was obviously increased via apelin-13, which was induced in a time- and dose-dependent manner in HUVECs. However, apelin-36 could not induce HUVEC proliferation and up-regulate PCNA and cyclinD1 expression.

Indeed, it had been reported that the relative potency of apelin peptides varies between experimental systems. For example, it had been shown that apelin-13 exhibited much stronger activity than apelin-36 in the cardiovascular system [34], whereas apelin-36 was the most potent inhibitor of HIV infection of cells *in vitro* [35]. Previous studies reported that apelin regulated the vascular tone *in vivo*, causing a decrease in blood pressure or vasodilation of resistance vessels [24]. *In vitro*, apelin caused vasodilation of human vessels on the endothelium in a nitric oxide-dependent manner [36]. Also, apelin caused vasoconstriction in the absence of a functional endothelium in human saphenous veins [37]. Since the biology and pathobiology of VECs were governed by the activity of a transcription factor network and cardiovascular activity regulating substances, it was also probably a negative feedback mechanism that maintained vascular homeostasis. ATRA not only induced prodifferentiation factors, such as RARα, but also up-regulated proproliferation factors, such as apelin. The hyperplasia of VECs was a key event in pathogenic vascular injuries, such as hypertension, atherosclerosis, and restenosis. Apelin and its active forms (apelin-36 and apelin-13) might have distinct biological activities that are necessary to regulate proliferation and anti-apoptosis in VECs. However, ATRA and RARα have effects on promoting differentiation and inhibiting proliferation in VECs.

Thus, the present study described a novel mechanism of apelin regulation by ATRA and RARα, leading to an increased understanding of the biological functions of ATRA and apelin during VEC proliferation modulation. Therapeutic manipulation of apelin represents a novel and potentially exciting therapeutic target especially in vascular disease.

## Supporting information

**Figure S1:** HUVEC proliferation induced by apelin-13(A,B) or apelin-36(C,D) was detected by the bromodeoxyuridine (BrdU) incorporation assay.

**Figure S2:** The effect of apelin-13 and apelin-36 on HUVECs proliferation-markered gene expression HUVECs were treated with apelin-13(A,B) or apelin-36(C,D) for various times or with different doses for 24 h. Crude proteins were extracted from the treated cells and then subjected to western blotting with anti-PCNA-actin, or anti-cyclinD1 antibodies. β-actin was used as a loading control.

## Abbreviations

ApoE: Apolipoprotein E
ATRA: all-*trans* retinoic acid
DMEM: Dulbecco’s modified Eagle’s medium
GAPDH: glyceraldehyde-3-phosphate dehydrogenase
HE: Hematoxylin–Eosin
HUVEC: human umbilical vein endothelial cell
pAd: plasmids adeovirus
PAH: pulmonary arterial hypertension
PCNA: proliferating cell nuclear antigen
qRT-PCR: quantitative reverse-transcription PCR
RAR: retinoic acid receptor
RARE: retinoic acid response element
RT: room temperature
RXR: retinoid X receptor
SD: Sprague–Dawley
si-NS: nonspecific siRNA
si-RARα: siRNA against RARα
TNF: tumor necrosis factor
VEC: vascular endothelial cell
