# Supplementary material for: The mechanism of all-trans retinoic acid in the regulation of apelin expression in vascular endothelial cells
Source: Biosci Rep. 2017 Dec 12;37(6):BSR20170684. doi: 10.1042/BSR20170684 (PMC5725614; doi:10.1042/BSR20170684)
Supplement: Supplementary file 1 [file bsr20170684_Supp1.pdf]

The mechanism of all-trans retinoic acid in the regulation of apelin expression in vascular endothelial cells

SHI HONGYUN, YUAN LANHUI, YANG HUIBIN, ZANG AIMIN*
Department of Oncology, the Affiliated Hospital of Hebei University, Baoding071000, Hebei, China
*Corresponding author: ZANG AIMIN
Department of Radiotherapy, the Affiliated Hospital of Hebei University,
No. 212 Yuhua East Road, Baoding 071000, Hebei, China
Tel: +86-15930487802
Fax: +86-21-64085875
E-mail: hyshi2015@163.com

Materials and Methods

Cell proliferation assay

HUVEC proliferation assays were performed with the bromodeoxyuridine (BrdU) Cell Proliferation Assay kit (Millipore) according to the manufacturer's recommendations. HUVECs were labeled for 6 h with BrdU prior to the termination of apelin-13 or apelin-36 (Sigma) incubation. Optical density readings were performed at 450nm. All groups were evaluated in a minimum of three separate wells per experiment.

Western blotting

Cells were treated with apelin-13 or -36 for various times or at different doses and then harvested with 150 mmol/L NaCl, 50 mmol/L Tris-HCl (pH7.5), 1% NP-40, 0.5% sodium deoxycholic acid and complete protease inhibitor mixture tablets (Roche Applied Science, Basel,Switzerland). Crude proteins were extracted from HUVECs, resolved by SDS-PAGE and transferred onto a polyvinylidene difluoride membrane (Millipore, Billerica, MA, USA). Membranes were blocked with 5% milk in Tris-buffered saline with Tween 20 (TTBS) for 2 h at 37°C and then incubated

overnight at 4°C with the following primary antibodies: 1:500 rabbit anti-PCNA (Santa Cruz, CA, USA), 1:1000 rabbit anti-cyclinD1 (Abcam, USA) and 1:1000 mouse anti-β-actin (Santa Cruz, CA, USA). After incubation with the appropriate secondary antibody, the immunoreactive signal of antibody-antigens were visualized using the Chemiluminescence Plus Western Blot analysis kit (Santa Cruz).

Results

Figure S1 HUVEC proliferation induced by apelin-13 and apelin-36

Apelin-13 could markedly induce HUVEC proliferation in a time- and dose-dependent manner (Figures S1A and 1B) as confirmed by BrdU incorporation experiments. Compared with apelin-13, apelin-36 could not significantly induce VSMC proliferationin a time- and dose-dependent manner (Figures 1C and 1D).

Figure S2 The effect of apelin-13 and apelin-36 on HUVEC proliferation-markered gene expression

we also investigated the effect of apelin-13 and apelin-36 on HUVEC proliferation. From Figure S2A-2B, the expression of the HUVEC proliferation-related genes PCNA and cyclinD1 was markedly up-regulated in a time- and dose-dependent manner. However, Compared with apelin-13, the expression of HUVECs proliferation-related genes PCNA and cyclinD1 has little changed compared with untreated HUVECs (Supplementary material Figure S2C and 2D). These results suggested that apelin isoforms apelin-13 and apelin-36 exhibit distinct pro-proliferation in HUVECs.

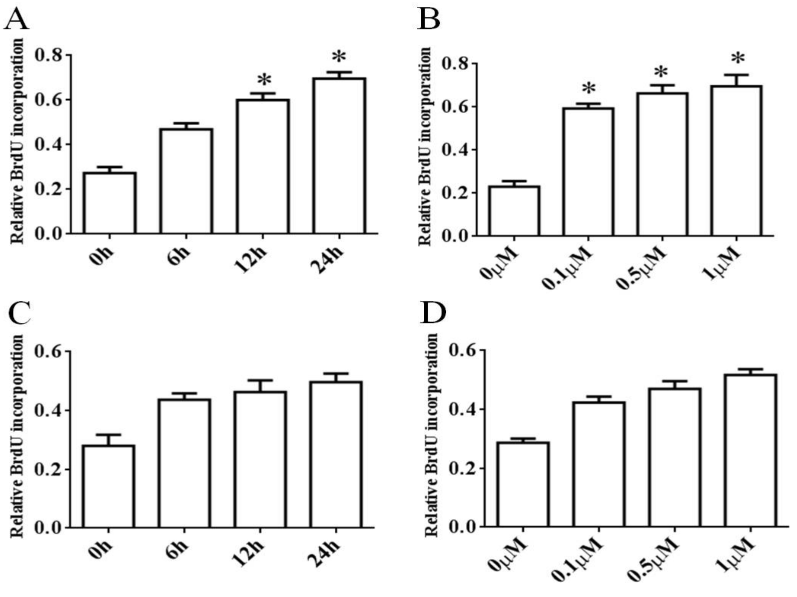

Figure S1

HUVEC proliferation induced by apelin-13(A,B) or apelin-36(C,D) was detected by the bromodeoxyuridine (BrdU) incorporation assay.

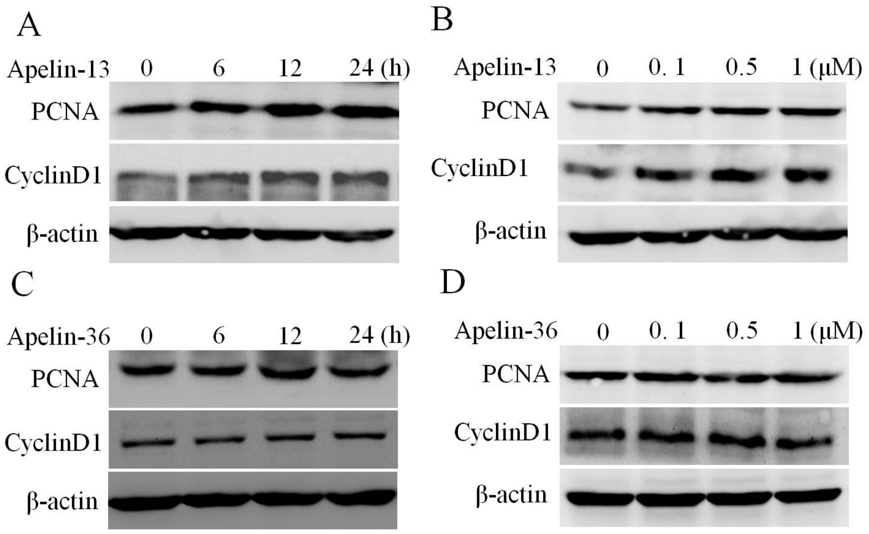

Figure S2 The effect of apelin-13 and apelin-36 on HUVECs proliferation-markered gene expression

HUVECs were treated with apelin-13(A,B) or apelin-36(C,D) for various times or with different doses for 24 h. Crude proteins were extracted from the treated cells and then subjected to western blotting with anti-PCNA-actin, or anti-cyclinD1 antibodies. β-actin was used as a loading control.
